# Prognostic Models to Trigger Palliative Care Referrals in Heart Failure: A Scoping Review

**DOI:** 10.1016/j.cjco.2026.06.011

**Published:** 2026-07-03

**Authors:** Klara Whalen, Hilary Bews, James W. Tam, Samuel Quan, Jana Pilkey

**Affiliations:** a Max Rady College of Medicine, University of Manitoba, Winnipeg, Manitoba, Canada; b Section of Palliative Medicine, Department of Internal Medicine, Max Rady College of Medicine, University of Manitoba, Winnipeg, Manitoba, Canada; c Section of Cardiology, Department of Internal Medicine, Max Rady College of Medicine, University of Manitoba, Winnipeg, Manitoba, Canada; d Section of Geriatric Medicine, Department of Internal Medicine, Max Rady College of Medicine, University of Manitoba, Winnipeg, Manitoba, Canada

**Keywords:** scoping review, heart failure, prognostication, six months, palliative

## Abstract

Accurate prognostication in heart failure (HF) is challenging, due to its nonlinear course. This course impacts the timing of goals of care conversations and referrals to palliative care. Consequently, prognostication may contribute to misunderstandings that can lead to poor end-of-life experiences for patients. As many hospice and palliative care programs have prognostic referral criteria of 6 months or less, we aimed to identify, compare, and synthesize prognostication models that would inform decision-making for palliative consultation and referrals. A scoping review was undertaken to map and characterize prognostic models that predict 6-month mortality in HF patients. An iterative literature search was conducted using PubMed, Embase, Emcare, Google Scholar, and Ovid MEDLINE, supplemented by hand searching, to identify applicable articles. Twenty studies from cardiology, palliative care, and geriatric medicine disciplines were given priority for inclusion in data charting and synthesis. Most models demonstrated moderate-to-high risk of bias and applicability concerns. The models were developed in diverse clinical settings with mortality timeframes ranging from in-hospital death to 6 months. Four common themes for prognostic indicators emerged across the disciplines: age, functional status, comorbidities/organ involvement, and prognostic awareness. Over time, discipline-specific scores have evolved to become more alike, to better address the unique needs of the advanced HF population. Papers differed widely in patient population, goals of prognostication, and applicability for informing palliative or hospice referrals. Developing more unified, multidisciplinary prognostic approaches could have value in supporting timely goals of care conversations, and palliative care involvement, for HF patients.

## Central Illustration

**Figure:**
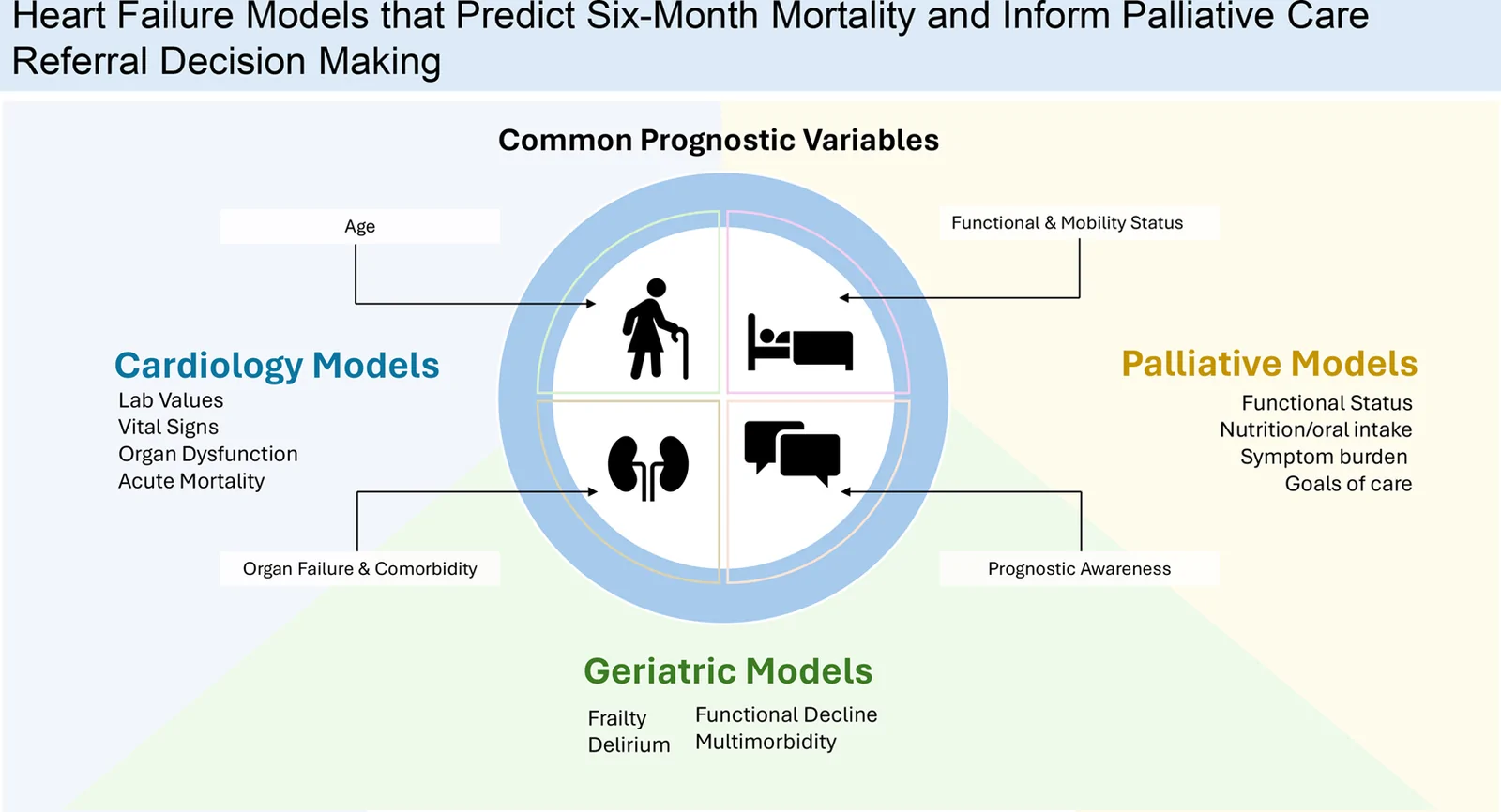

## Résumé

Établir un pronostic précis dans l’insuffisance cardiaque (IC) est difficile en raison de son évolution non linéaire. Cela influe sur le moment choisi pour aborder les objectifs de soins et pour orienter les patients vers les soins palliatifs. Par conséquent, cela peut contribuer à des malentendus susceptibles d’entraîner une mauvaise expérience de fin de vie pour les patients. Comme de nombreux programmes de soins palliatifs utilisent comme critère d’orientation un pronostic vital de six mois ou moins, nous avons cherché à identifier, comparer et synthétiser les modèles pronostiques susceptibles d’éclairer la prise de décision concernant la consultation et l’orientation vers les soins palliatifs. Une revue exploratoire a été menée afin de recenser et de caractériser les modèles pronostiques permettant de prédire la mortalité à six mois chez les patients atteints d’IC. Une recherche bibliographique itérative a été effectuée à l’aide de PubMed, Embase, Emcare, Google Scholar et Ovid MEDLINE, complétée par une recherche manuelle, afin d’identifier les articles pertinents. Vingt études relevant de la cardiologie, des soins palliatifs et de la gériatrie ont été retenues en priorité pour être incluses dans la synthèse et la présentation des données. La plupart des modèles présentaient un risque de biais modéré à élevé et soulevaient des préoccupations quant à leur applicabilité. Ces modèles ont été développés dans divers contextes cliniques, avec des horizons temporels de mortalité allant du décès à l’hôpital à six mois. Quatre thèmes communs aux indicateurs pronostiques se sont dégagés dans l’ensemble des disciplines : l’âge, l’état fonctionnel, les comorbidités/atteinte d’organes et la prise de conscience du pronostic. Au fil du temps, les scores spécifiques à chaque discipline ont évolué pour se rapprocher les uns des autres, afin de mieux répondre aux besoins particuliers de la population atteinte d’IC avancée. Les publications présentaient de grandes disparités en termes de population de patients, d’objectifs de pronostic et d’applicabilité pour orienter vers des soins palliatifs ou des centres de soins de fin de vie. Le développement d’approches pronostiques plus unifiées et multidisciplinaires pourrait s’avérer utile pour favoriser des discussions en temps opportun sur les objectifs de soins et l’implication des soins palliatifs chez les patients atteints d’IC.

Heart failure (HF) is a leading cause of hospitalization, affecting 750,000 Canadians^1^ and 64 million people worldwide.^2^ Despite treatment advances, approximately 50% of patients die within 5 years of diagnosis.^3^^,^^4^ Readmission rates in HF are approximately 20%, and readmission most frequently occurs within 3 months of discharge.^5^^,^^6^ HF follows an unpredictable trajectory, marked by chronic decline, intermittent exacerbations, and occasional short-term fluctuations in functional status, making prognostication challenging.^3^^,^^7^^,^^8^ This prognostic uncertainty contributes to delay of conversations regarding care goals.^9^ Subsequently, high numbers of HF patients die in the hospital, despite literature suggesting that most patients prefer to die at home.^10^

Further contributing to the prognostic uncertainty and conversation delay is the relative paucity of literature predicting death within 6 months, a common cutoff for hospice and palliative care program consultation and enrollment. Widely used prognostic HF tools, such as the Seattle Heart Failure Model (SHFM)^11^ and the **M**eta-**A**nalysis **G**lobal **G**roup **i**n **C**hronic Heart Failure (MAGGIC) score,^12^ were not derived, or validated, for predicting a prognosis of 6 months or less. They are therefore limited in their usefulness in helping clinicians determine the appropriate timing for hospice or palliative care referrals.

Despite evidence of the benefit of consultation and referral to palliative care and hospice programs,^13^ HF patients are often under-referred.^14^ When referrals do happen, patients are older, have lower functional status, and are often already critically ill.^14^ Palliative care for advanced illness is a class I recommendation in HF guidelines,^15^ yet there no standardized referral triggers have been established.^16^ Robust, user-friendly prognostic tools that estimate short-term mortality may help improve palliative care consultation and hospice referral for HF patients as they approach their end-of-life period.

## Objectives and Focus of Review

A scoping review was undertaken to identify, compare, and synthesize prognostic models relevant to short-term mortality and palliative care referral decision-making in HF, with a particular emphasis on mortality horizons of 6 months or less. A 6-month timeframe was selected for this review because it is a common and clinically relevant threshold that can facilitate access to additional supports through referral to palliative care programs and/or hospice enrollment.^17^^,^^18^ Although other short-term outcomes are also clinically relevant, mortality is often a hard cutoff for many different palliative care programs, and ambiguous clinical estimates of mortality may preclude palliative care or hospice involvement. This context makes the ability to predict 6-month mortality very important for all clinicians who provide care for advanced HF patients.

Currently, 3 separate bodies of literature have looked at prognosis in HF—including literature published in cardiology, palliative care, geriatric, and general medicine journals. Within the existing literature, comparisons of prognostic models have been made within individual disciplines, but until now, cross-disciplinary comparisons, using the lens of referral to palliative and hospice programs, have been limited.

### Primary objective

The objective of this scoping review was to map the existing literature on prognostic models in HF. We hoped to identify key prognostic indicators, common themes, and methodological approaches, examining how these models are applied in relation to palliative care referral.

### Secondary objective

A secondary objective was to compare how prognostication is conceptualized across disciplines. This conceptualization is reflected by comparison of the selection and emphasis of prognostic indicators within the included models.

## Methods

This scoping review was conducted and reported in accordance with the Preferred Reporting Items for Systematic Reviews and Meta-Analyses extension for Scoping Reviews (PRISMA-ScR) guidelines.^19^ The review follows the methodological framework proposed by Arksey and O’Malley,^20^ and refined by Levac et al.^21^ The review process included identifying the research question, establishing eligibility criteria, conducting a comprehensive literature search, selecting and charting relevant studies, and synthesizing findings descriptively and thematically. Ethics approval and patient consent were waived by the local human research ethics board, due to the nature of the study.

### Identifying the research question

This review aimed to examine prognostic scoring systems used in HF across disciplines, with a focus on their ability to predict 6-month mortality and inform the timing of palliative care consultation and/or hospice referral. A preliminary exploration of the literature confirmed sufficient heterogeneity to justify a scoping review approach. To help focus the review, the research team considered the 3 following questions:

1. Which prognostic scoring systems predict death for HF patients within 6 months, and how might they inform timely palliative and hospice care referrals?
2. What similarities and differences exist in prognostic approaches across disciplines?
3. How have the prognostic approaches changed over time?

### Eligibility criteria

Eligibility criteria were structured using the population–concept–context approach recommended for scoping reviews.^22^ The **population** included adults aged ≥ 18 years with a confirmed diagnosis of HF, determined by clinical diagnosis, or Framingham criteria.^23^ All studies involving HF were eligible, including studies with acute or chronic HF, and studies with preserved or reduced ejection fraction.

The **concept** of interest was prognostication in HF; specifically, models, or scoring systems designed to estimate 6-month mortality. Six-month mortality was chosen because many hospice and palliative care programs are designed to focus on the last 6 months of life. Variation across disciplines and prognostic settings was anticipated, and all were considered clinically relevant because palliative care supports patients across care settings, including hospitals, homes, and hospices.

Studies were eligible if they developed, validated, or evaluated a HF prognostic model that included all-cause mortality with a prognosis of 6 months or less. Studies examining mortality as a secondary outcome were included if mortality was explicitly incorporated into model development or validation. Studies that were focused exclusively on symptom burden, quality of life, healthcare utilization, or nonmortality outcomes were excluded. When multiple publications described the same prognostic model, the most comprehensive derivation and validation reports were reviewed to avoid duplicate representation.

The **context** encompassed all clinical settings in which HF prognostication might occur. Emergency departments, hospital wards, critical care units, coronary care units, outpatient clinics, and geriatric units were included. No geographic or healthcare system restrictions were applied, to maintain as broad an applicable as possible, acknowledging that palliative consultation or hospice referrals can be triggered in many different settings. All papers published up until the search date could be included.

### Literature search

Assisted by a medical librarian, a comprehensive search strategy was developed. PubMed, Embase, Emcare, Google Scholar, and Ovid MEDLINE(R) ALL, from 1946 to February 19, 2025 (the date of the search), were searched to identify scholarly peer-reviewed articles and grey literature. The search strategy was designed to identify prognostic models relevant to HF, palliative care, hospice care, and end-of-life decision-making. Search terms included combinations of concepts related to palliative care, hospice care, prognostication, prediction models, scoring systems, and HF. The core search strategy included the following terms: (palliati∗ or hospice∗ or terminal∗) adj3 (score or scores or scoring or scale∗ or index or indices or predict∗ or prognos∗)) and (heart or cardi∗ or myocardi∗) adj2 (fail∗ or decompensat∗)). Database-specific syntax modifications were made as required.

The search was intentionally broad to capture literature from cardiology, palliative care, geriatric medicine, and related disciplines. Cardiology and palliative care search strategies were developed separately and were combined to identify articles addressing HF prognostication within the context of palliative or end-of-life care. An iterative snowball approach, including reference list screening and expert consultation, identified additional studies.

The search identified 643 articles. After removal of 253 duplicates, 390 records underwent title and abstract screening. Additional records were identified through hand-searching and expert consultation. The study selection process is summarized in Figure 1.

**Figure 1:**
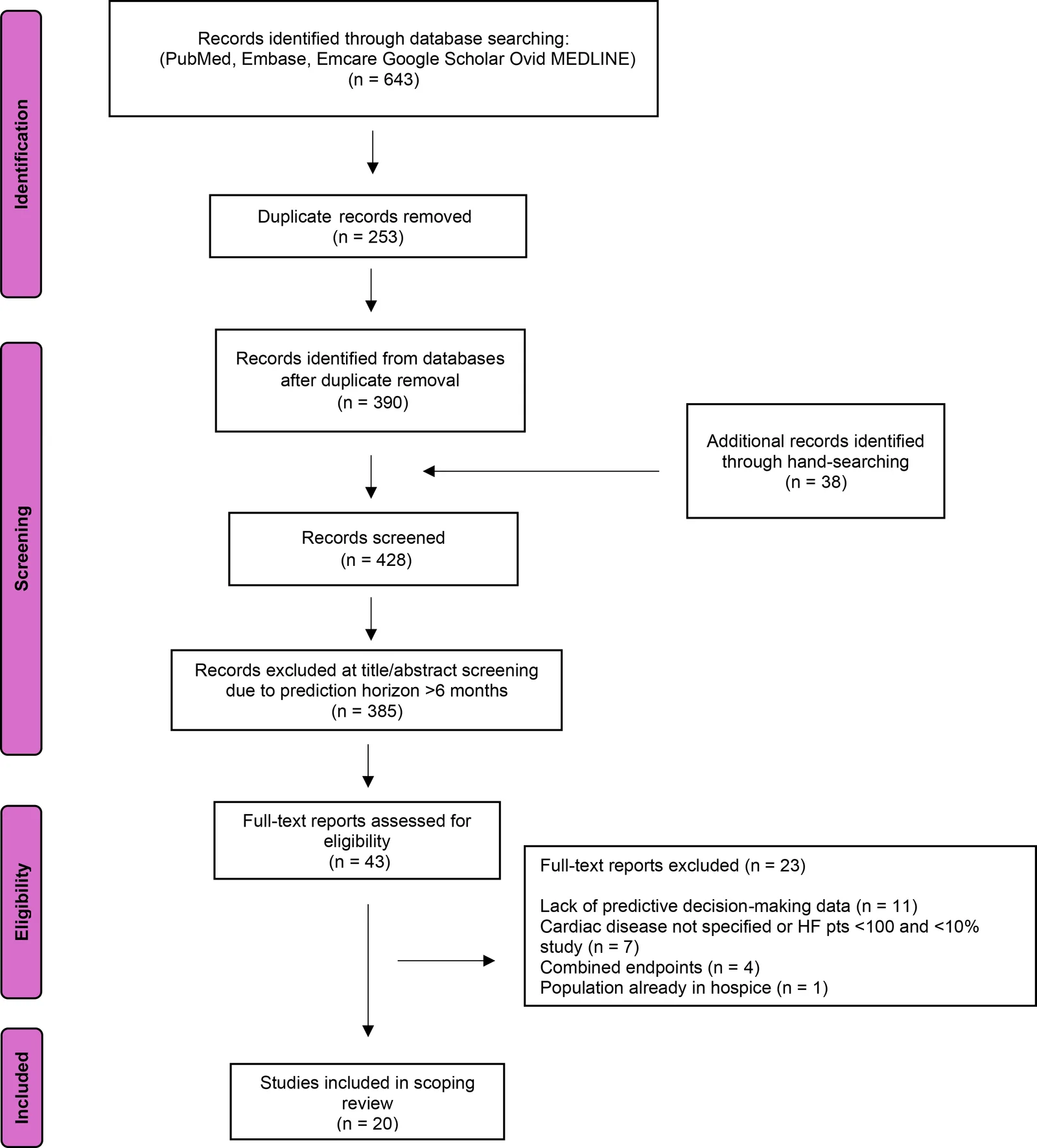
Preferred Reporting Items for Systematic Reviews and Meta-Analyses extension for Scoping Reviews (PRISMA-ScR) flow diagram of study selection. Records screened for inclusion based on a prediction horizon of ≤ 6 months. HF, heart failure; pts, patients.

### Study selection

A total of 428 titles and abstracts were screened independently by members of the research team, to identify studies describing prognostic models in adult patients with HF. Given the exploratory nature of the review, and the heterogeneity of prognostic approaches across disciplines, potentially relevant studies were retained for full-text review when eligibility was uncertain.

A total of 43 articles underwent full-text review by 2 independent reviewers. Studies were eligible if they evaluated prognostic models, risk scores, or prediction tools in adult patients with HF and reported mortality outcomes relevant to short-term prognostication and palliative care referral decision-making. Although the primary focus of the review was 6-month mortality, studies predicting shorter mortality timeframes were also considered if they potentially could be used to inform clinical decision-making regarding referrals to palliative and hospice care in advanced HF populations.

Studies were excluded if HF populations were not clearly defined, or if HF patients represented less than 10% of the study population or fewer than 100 patients. They were also excluded if mortality outcomes were only reported as part of composite endpoints, if populations were already enrolled in hospice programs, if insufficient prognostic data were available to permit meaningful interpretation, or if the intent of the article was to predict adverse events, resource utilization, or treatment requirements. Disagreements were resolved through discussion and consensus. Following full-text review, 20 studies were included in the final synthesis. To promote consistent application of the eligibility criteria, inclusion and exclusion decisions were documented during full-text review, and reasons for exclusion were recorded and summarized in the PRISMA-ScR flow diagram (Fig. 1).

### Charting and analysis of data

Data were collected using a standardized form, collaboratively developed by the research team. Data collected included study population characteristics, care settings, prognostic variables, outcome measures, mortality rates (overall and stratified), derivation and validation cohorts, and evaluation of the models, including calibration and discrimination analyses. Three reviewers initially charted the data, discussed the results, and continuously updated the data-charting form, in an iterative process.

As prognostic model performance is dependent on methodology and applicability, the **P**rediction Model **R**isk of **B**ias **As**sessment **T**ool (PROBAST)^24^ framework was used to descriptively map and evaluate the studies in accordance with the **T**ransparent **R**eporting of a Multivariable Prediction Model for **I**ndividual **P**rognosis **o**r **D**iagnosis (TRIPOD) guidelines for reporting prediction models.^25^ Two independent reviewers completed the PROBAST for the 20 studies, assessing risk of bias and applicability across the domains of participant selection, predictors, outcomes, and analysis,^24^ determining the model’s ability to support palliative care or hospice referral decisions. Discrepancies in assigned PROBAST scores between the reviewers were discussed by the team to obtain consensus.

Risk-of-bias and applicability assessments are summarized in Table 1. Overall, risk of bias was frequently identified across studies, and the analysis domain was the most common source of concern. Common methodological limitations included incomplete reporting of model development and validation procedures, limited assessment of calibration, and a lack of external validation. Applicability was also a source of concern. Although most studies evaluated clinically relevant HF populations and used predictors and outcomes applicable to prognostic assessment, most were not intended to inform palliative care referral decision-making. Applicability concerns were most common among studies with very restricted patient populations.

**Table 1:** Risk of bias and applicability assessment

Score Name (Study)	Risk of bias				Applicability			Overall	
	1. Participants	2. Predictors	3. Outcome	4. Analysis	1. Participants	2. Predictors	3. Outcome	Risk of bias	Applicability
**EFFECT**, Lee et al.^40^ (2003)									
**OPTIME-CHF,** Felker et al.^27^ (2004)									
**ADHERE**, Fonarow et al.^26^ (2005)									
**ESCAPE**, O’Connor et al.^30^ (2010)									
**GWTG-HF,** Peterson et al.^28^ (2010)									
**EHMRG**, Lee et al.^41^ (2012)									
**APACHE-HF,** Okazaki et al.^43^ (2014)									
**ELAN-HF**, Salah et al.^29^ (2019)									
**MEESI**, Wussler et al.^42^ (2019)									
**EPICTER,** Romero-Correa et al.^31^ (2022)									
**SOB-ASAP**, Shiraishi et al.^45^ (2020)									
**PPI**, Martin et al.^34^ (2013)									
Snow et al.^35^ (2016)									
**PALIAR**, Bernabeu-Wittel et al.^33^ (2014)									
**PPI**, Sumi et al.^32^ (2019)									
**IPOS-ESC,** Hamatani et al.^44^ (2022)									
**In-hospital mortality risk score**, Abarca et al.^39^ (2018)									
**MPI**, Huynh et al.^37^ (2008)									
**Impact of delirium**, Sato et al.^36^ (2017)									
**PNI-ESC**, Candeloro et al.^38^ (2020)									

Low risk of bias/low concern of applicability.

Unclear risk of bias/ unclear applicability.

High risk of bias/high concern of applicability.

ADHERE, **A**cute **D**ecompensated **He**art Failure National **Re**gistry; APACHE-HF, **A**cute **P**hysiology **a**nd **C**hronic **H**ealth **E**valuation–**H**eart **F**ailure; EFFECT, **E**nhanced **F**eedback **f**or **E**ffective **C**ardiac **T**reatment; EHMRG, **E**mergency **H**eart Failure **M**ortality **R**isk **G**rade; ELAN-HF, **E**uropean Co**l**laboration on **A**cute Decompe**n**sated **H**eart **F**ailure; EPICTER, registry/study Name: Studio para Identificar Pacientes con Insuficiencia Cardiaca Terminal (roughly: “Study to Identify Patients with Terminal Heart Failure”); ESCAPE, **E**valuation **S**tudy of **C**ongestive Heart Failure **a**nd **P**ulmonary Artery Catheterization **E**ffectiveness; GWTG-HF, **G**et **w**ith **t**he **G**uidelines–**H**eart **F**ailure; IPOS-ESC, **I**ntegrated **P**alliative Care **O**utcome **S**cale – **E**uropean **S**ociety of **C**ardiology; MEESSI, Multiple Estimation of Risk Based on the Spanish Emergency Department Score in Patients with Acute Heart Failure; MPI, Multidimensional Prognostic Index; OPTIME-CHF, **O**utcomes of a **P**rospective **T**rial of **I**ntravenous **M**ilrinone for **E**xacerbations of **C**hronic **H**eart **F**ailure; PALIAR, (from the Spanish palliative care program/registry name [not an acronym]); PNI-ESC, **P**rognostic **N**utritional **In**dex – **E**mergency **S**ervices/**C**are; PPI, **P**alliative **P**rognostic **I**ndex; SOB-ASAP, (Components-based acronym): **s**odium, **o**ld age, **b**lood urea nitrogen (B), **a**lbumin, **s**ystolic blood pressure, **a**cute heart failure **p**eptide (BNP/NT-proBNP).

Full research team meetings occurred weekly during the charting process to ensure that charting was accurate and remained relevant. Papers were also mapped according to population, contextual relevance, discipline of the publishing journal, and total and stratified mortality (Table 2). Prognostic variables were collected from each paper. The indicators were presented in tabular form, to visually show which individual indicators were most commonly present. Age, functional status, and indicators of cardiac and other organ dysfunction comorbidities were common. Prognostic awareness was a unique, but uncommon, prognostic factor (Table 3). These indicators were subsequently placed into 4 groups of like variables for further analysis.

**Table 2:** Summary of papers

Study	Paper name	Score name	Inclusion/exclusion criteria	Population & total mortality	Risk-stratified mortality	End-point	Relevant statistics
Lee et al.^40^ (2003)	Predicting Mortality Among Patients Hospitalized for Heart Failure Derivation and Validation of a Clinical Model	**EFFECT**	**Inclusion**: Met Framingham HF criteria **Exclusion**: Developed HF after admission Patients transferred from another acute care facility Age ≥ 105 Y Nonresidents Individuals with invalid health cards	n = 4031 Derivation cohort n = 2624 Validation cohort n = 1407 HFpEF and HFrEF inpatients LVEF ≤ 40%: Derivation cohort = 52.8% Validation cohort = 47.7% Total mortality (derivation/validation): In hospital: 8.9%, 8.9% 30 d: 10.7%, 10.4%	Mortality by low/high group: 30-d = 0.4%, 59%	30-d mortality	Calibration: No calibration reported Goodness of fit: Hosmer-Lemeshow X^2^ statistic *P* > 0.05 for all models Discrimination: C statistic: Derivation 30-d = 0.80 Validation 30-d = 0.79
Felker et al.^27^ (2004)	Risk Stratification After Hospitalization for Decompensated Heart Failure	**OPTIME- CHF**	**Inclusion:** Systolic dysfunction **Exclusion:** (From **OPTIME- CHF)** Inotropic therapy Active ischemia within 3 mo SBP < 80 mm Hg Creatinine > 265 umol/L Unstable arrythmias	n = 949 HFrEF inpatients Total mortality not reported	Mortality by points: 124 = 2% 149 = 4% 163 = 6% 174 = 8% 182 = 10% 208 = 20% 225 = 30%	60-d mortality	Calibration: calibration plot Goodness of fit: not reported Discrimination: C statistic: 0.77
Fonarow et al.^26^ (2005)	Risk Stratification for In-Hospital Mortality in Acutely Decompensated Heart Failure Classification and Regression Tree Analysis	**ADHERE**	**Inclusion**: In-hospital ADHF Age ≥ 18 Y Included multiple hospitalizations of same patient	n = 65,275 Derivation cohort n = 33,046 Validation cohort n = 32,229 HFpEF and HFrEF inpatients LVEF ≥ 40%: Derivation cohort = 44% Validation cohort = 47% Total mortality = 4.1%	Mortality by quintile: Group 1= 2.1% Group 2 = 4.0% Group 3 = 6.3% Group 4 = 13.2% Group 5 = 20.0%	In-hospital mortality	Calibration: no calibration reported Goodness of fit: not reported Discrimination: C statistic: Derivation = 0.687 Validation = 0.668
O’Connor et al.^30^ (2010)	Triage After Hospitalization with Advanced Heart Failure: The ESCAPE (Evaluation Study of Congestive Heart Failure and Pulmonary Artery Catheterization Effectiveness) Risk Model and Discharge Score	**ESCAPE** in hospital **&** discharge model	**Inclusion:** Patients meeting the following inclusion criteria within the past year: Urgent ED visit in preceding month > 160 mg of furosemide daily ACE-I and diuretic use ≥ 3 mo LVEF ≤ 30% SBP ≤ 125 mm Hg At least 1 sign and 1 symptom of congestion **Exclusion:** Serum creatinine > 309 mmol/L Prior use of dobutamine/dopamine > 3 μg/kg/min Prior use of milrinone	n = 423 Total mortality = 18.7%	Mortality discharge score: 0 points—7% 1 points—10% 2 points—16% 3 points—26% 4 points—44% 5 points—80% 6 points—75% ≥ 7 points—100%	6-mo mortality	Calibration: calibration plot Goodness of fit: not reported Discrimination: C statistic: Escape model = 0.76 Discharge model = 0.74
Peterson et al.^28^ (2010)	A Validated Risk Score for In-Hospital Mortality in Patients With Heart Failure from the American Heart Association Get With the Guidelines Program	**GWTG-HF**	**Inclusion:** Inpatients with HF defined by treating clinician's diagnosis **Exclusion:** No diagnosis of HF Transferred to different acute care facility Discharge date invalid Data missing for discharge status Data missing for LVEF	n = 39,783 Derivation cohort n = 27,850 Validation cohort n = 11,933 Total mortality = 2.86%	Mortality by decile: 1—0.4% 2—0.7% 3—1.0% 4—1.3% 5—1.6% 6—2.1% 7—2.5% 8—3.3% 9—4.6% 10—9.7%	In-hospital mortality	Calibration: calibration plot Goodness of fit: Hosmer-Lemeshow test Predicted mortality *P* = 0.189, Observed mortality *P* = 0.604 Discrimination: C statistic: Derivation = 0.75 Validation = 0.75
Lee et al.^41^ (2012)	Prediction of Heart Failure Mortality in Emergent Care: A Cohort Study	**EHMRG**	**Inclusion**: Met Framingham HF criteria HF patients presenting to the ED **Exclusion**: Patients who were palliative Patients with DNR orders before arrival Transfers from another acute care hospital Dialysis-dependent patients	n = 12,591 Derivation cohort n = 7433 Validation cohort n = 5158 LVEFs not reported	Mortality by quintiles (Q): Q1, Q2 = 0.3% Q3 = 0.7% Q4 =1.9% Q5a= 3.5% Q5b = 8.2%	1-wk mortality	Calibration: no calibration reported Goodness of fit: Hosmer-Lemeshow 2.99; *P* -0.935 Discrimination: C statistic: Derivation = 0.805 Validation = 0.826
Okazaki et al.^43^ (2014)	New Scoring System (APACHE-HF) for Predicting Adverse Outcomes in Patients with Acute Heart Failure: Evaluation of the APACHE II and Modified APACHE II Scoring Systems	**APACHE-HF**	**Inclusion**: AHF patients admitted to ICU with 1 of: Projectile O_2_ inhalation (including mechanical support) Inotrope/mechanical support, low BP Diuretics Met Framingham HF criteria & NYHA functional class III or IV **Exclusion:** Patients with ACS	n = 824 HFpEF and HFrEF ICU patients LVEF ≥ 40% = 36% Total mortality = 8.98%	Mortality discharge score: 0–3 = 0% 4–6 = 2.2% 7–9 = 4.6% 10–12 = 14.3% > 12 = 32.7%	90-d mortality	Calibration: no calibration reported Goodness of fit: not reported Discrimination: C statistic = 0.779
Salah et al.^29^ (2019)	External Validation of the ELAN-HF Score, Predicting 6- Month All-Cause Mortality in Patients Hospitalized for Acute Decompensated Heart Failure	**ELAN-HF**	**Inclusion:** Patients admitted with clinically validated ADHF, discharged alive and had NT- proBNP measurements at admission and discharge, from 7 previous study cohorts **Exclusion**: No follow-up	n = 1301 HFpEF and HFrEF acutely decompensated Total mortality = 15%	Mortality by risk group: Derivation/Validation: Low = 3.6%, 7.0% Intermediate = 9.2%. 12.9% High = 23.5%, 23.4% Very high = 51.1%, 51.7%	6-mo mortality	Calibration: calibration plot Goodness of fit: not reported Discrimination: C-statistic = 0.78
Wussler et al.^42^ (2019)	External Validation of the MEESSI Acute Heart Failure Risk Score: A Cohort Study	**MEESSI**	**Inclusion:** Patients presenting to ED with acute dyspnea Met HF Framingham criteria **Exclusion:** Hemodialysis Missing complete data Lost to follow-up	n = 1247 Total mortality = 8.2%	Mortality by sextile: Q1—0% Q2—0.4% Q3—4.9% Q4—10.1% Q5—15% Q6—28.5%	30-d mortality	Calibration: calibration plot Goodness of fit: Hosmer-Lemeshow = 0.022 Risk overestimated in high-risk groups; after adjustment between predicted/observed: *P* = 0.23 Discrimination: C statistic = 0.80
Romero-Correa et al.^31^ (2022)	The EPICTER Score: A Bedside and Easy Tool to Predict Mortality At 6 Months in Acute Heart Failure	**EPICTER**	**Inclusion**: Inpatients with HF **Exclusion:** Lost to follow-up Lack of consent	n = 2848 Derivation cohort n = 1709 Validation cohort n = 1139 LVEF available in 972 patients: *All patients with LVEF <40% reported* HFpEF (59.3%) HFmEF (14.2%) HFrEF (26.5%) Total mortality = 26.4% Derivation cohort = 26.1% Validation cohort = 26.9%	Mortality by group: Low (0–3) = 5.7% Medium (4–9) = 17.8% High (10–16) = 35.1% Very high (17–25) = 53.8%	6-mo mortality	Calibration: no calibration reported Goodness of fit: not reported Discrimination: C statistic: Derivation= 0.74 Validation = 0.68
Shiraishi et al.^45^ (2020)	Derivation and Validation of Clinical Prediction Models for Rapid Risk Stratification for Time-Sensitive Management for Acute Heart Failure	**SOB-ASAP**	**Inclusion**: Met Framingham HF criteria Scheduled and unscheduled hospital visits and/or admissions (derivation cohort) Hospitalized through ED (validation cohort) **Exclusionfrom REALITY-AHF(Registry Focused on Very Early Presentation and Treatment in Emergency Department of Acute Heart Failure)** Patients presenting with ACS Validation cohort: Baseline NT-BNP levels < 300 pg/mL	n = 6033 Derivation cohort n = 4351 Validation cohort n = 1682 Mean EF: Derivation cohort = 43.5% Validation cohort = 46.3% Mortality Derivation cohort= 3.9% Validation cohort = 5.1%	Mortality by sextile: Q1, Q2 = <1% Q6 = 14.5%	In-hospital mortality	Calibration: calibration plot Goodness of fit: not reported Discrimination: C-statistic: Derivation = 0.82 Validation = 0.71
Martin et al.^34^ (2013)	Adaptation of Palliative Prognostic Index in Patients with Advanced Medical Conditions	**PPI** (Adaptation)	**Inclusion**: Patients from inpatient wards, home hospitalization, day units and outpatient clinics and 1 or more of: HF with dyspnea ≥ NYHA 3 Respiratory failure & baseline dyspnea Chronic renal failure; stage 4-5 Chronic liver disease; Child-Pugh > 7 Neurological disease with cognitive impairment	n: 1788 HF patients = 38.9% Total mortality = 37.5% Interim mortality 30 d = 15.8% 60 d = 22.8% 90 d = 28.1% 120 d = 31.7% 150 d = 37.5%	Mortality by quintiles: Q1 (> 0 points) = 13% Q2 (1–2 points) = 23% Q3 (2.5–9.5 points)= 39% Q4 (> 9.5 points) = 68%	6-mo mortality	Calibration: no calibration reported Goodness of fit: Hosmer-Lemeshow *P* = 0.037 Discrimination: C statistic = 0.69
Snow et al.^35^ (2016)	A Prognostic Indicator for Patients Hospitalized with Heart Failure		**Inclusion**: Patients with HF Patients from Medicare/Medicaid claims data (fee-for-service beneficiaries) **Exclusion**: Patients in Medicare Advantage program	n = 7508 HF patients = 100% Total mortality = 24.3%	Not provided	6-mo mortality	Calibration: no calibration reported Goodness of fit: Hosmer-Lemeshow *P* = 0.98 Discrimination: C statistic = 0.71
Bernabeu-Wittel et al.^33^ (2014)	Development of a Six-Month Prognostic Index in Patients with Advanced Chronic Medical Conditions: The PALIAR Score	**PALIAR**	**Inclusion**: Aged ≥ 18 years from in hospital informed consent One or more advanced conditions: HF Respiratory failure Chronic renal failure Chronic liver disease Chronic neurological disease **Exclusion**: Active neoplasm Lost to follow-up	N = 1778 Derivation n = 884 Validation n = 894 HF patients = 38.9% Deviation = 38% Validation = 39.7% Total mortality Derivation = 37.6% Validation = 37.7%	Mortality by risk scores: derivation/validation: 0 = 20%, 21.5% 3–3.5 = 33%, 30.5% 4–7.5 = 46.3%, 43% ≥ 7.5 = 67%, 61%	6-mo mortality	Calibration: calibration in the large Goodness of fit: Hosmer-Lemeshow *P* = 0.39 Discrimination: C statistic = 0.69
Sumi et al.^32^ (2019)	Prognostic Utility of Palliative Prognostic Index (PPI) for Prediction of 30-day and 1-year Outcome in Patients with ADHF	**PPI** (Prognostic Utility**)**	**Inclusion**: Patients with ADHF admitted to hospital	n = 764 patients	Mortality by risk group: High-PPI (≥ 6) /Low-PPI (< 6) 30-d = 7.9 / 2.0%, 1-Y = 20.0 / 12.7%	30-d mortality 1-Y mortality	Calibration: no calibration reported Goodness of fit: not reported Discrimination: C statistic = 0.720
Hamatani et al.^44^ (2022)	Comprehensive Symptom Assessment Using Integrated Palliative Care Outcome Scale in hospitalized Heart Failure Patients	**IPOS ESC**	**Inclusion**: Met Framingham criteria for HF **Exclusion**: Patients unable to fill out IPOS questionnaires: Unwillingness to respond Dementia Prior stroke Depressed level of consciousness Hard of hearing Death soon after admission Early discharge Worsening HF Incomplete IPOS response	n = 294 HF patients = 100% Mean LVEF = 44%	Worsening IPOS = 15.4% Without worsening IPOS = 7.77%	Mortality at median follow-up period of 238 d (range of 100-416 d)	Calibration: no calibration reported Goodness of fit: not reported Discrimination: C statistic not reported
Abarca et al.^39^ (2018)	In-hospital Mortality Risk Score for Very Old Patients Hospitalized with Decompensated Chronic Heart Failure	**In-Hospital Mortality Risk Score**	**Inclusion**: Age ≥ 80 Y Admission to geriatric acute care unit ADHF	n = 629	Very low risk = 3% Low risk = 4.6% Moderate risk = 9.5% High risk = 15.1% Very high risk = 36.3%	In-hospital mortality	Calibration: no calibration reported Goodness of fit: Hosmer-Lemeshow *P* = 0.185 Discrimination: C statistic = 0.77
Huynh et al.^37^ (2008)	Identification of Older Patients with Heart Failure Who May Be Candidates for Hospice Care: Development of a Simple Four-Item Risk Score	**MPI**	**Inclusion**: Diagnosis of HF: Definite clinical improvement to diuresis with either: Radiographic pulmonary congestion or Typical symptoms and signs of HF Age ≥ 70 Y **Exclusion**: From Original Paper: Residence outside catchment area Planned discharge to long-term-care Severe dementia or psychiatric illness Prognosis < 3 mo Refusal by patient or physician Logistic or discretionary reasons	n = 282 Mean LVEF = 42% HFpEF = 47% Total mortality = 15.2%	Number of risk factors: 0—3.7% 1—16.3% 2—41% ≥ 3—66.7%	6-moall-cause mortality	Calibration: no calibration reported Goodness of fit: not reported Discrimination: C statistic = 0.8
Sato et al.^36^ (2017)	The Impact of Delirium on Outcomes in Acute, Non-Intubated Cardiac Patients	**Impact of Delirium (score for delirium and 60-day mortality)**	**Included**: Cardiac patients admitted to ICU or ICCU age ≥ 65 Y **Excluded**: Surgical patients Mechanically ventilated patients ICU stay of ≤ 48 h Patients with coexisting infections or advanced cancer Patients unable to communicate	n = 163 HF patients = 45% ADHF 45%, ACS 40% (If ADHF related to ACS, then classified as ACS)	With delirium = 22.9% Without delirium = 3.9%	60-d mortality	Calibration: no calibration reported Goodness of fit: not reported Discrimination: C statistic = 0.843
Candeloro et al.^38^ (2020)	Prognostic Nutritional Index in Elderly Patients Hospitalized for Acute Heart Failure	**PNI - ESC**	**Inclusion**: ADHF **Exclusion**: Age < 65 Y Presence of sepsis Severe liver cirrhosis Lack of informed consent	n = 344 NYHA II = 16.9% NYHA III = 54.7% NYHA IV = 28.5% Cardiovascular death = 41%	Low PNI (≤ 34) / High PNI (> 34): Overall = 29.1%, 12.9% In-hospital = 12.7%, 2.6% Post-Discharge = 16.4%, 10.3%	6-mo all-cause mortality	Calibration: no calibration reported Goodness of fit: not reported Discrimination: C statistic not reported

C statistic used to represent C statistic, C index, area under the curve (AUC), and receiver operating characteristic (ROC).

ACE-I, angiotension-converting enzyme inhibitor; ACS, acute coronary syndrome; ADHF, acute decompensated heart failure; AHF, acute heart failure; AUC, area under the curve; BNP, brain natriuretic peptide; BP, blood pressure; CHF, congestive heart failure; DNR, do not resuscitate; ED, emergency department; EF, ejection fraction; HF, heart failure; HFpEF, heart failure with preserved ejection fraction; HFrEF, heart failure with reduced ejection fraction; ICCU, intensive cardiac care unit; ICU, intensive care unit; LVEF, left ventricular ejection fraction; NT-proBNP, N-terminal pro-B-type natriuretic peptide; NYHA, New York Heart Association functional class; OP, original paper; PND, paroxysmal nocturnal dyspnea; PNI, Prognostic Nutritional Index; PPI, Palliative Prognostic Index; RVR, rapid ventricular response; SBP, systolic blood pressure.

Score names:

ADHERE, **A**cute **D**ecompensated **He**art Failure National **Re**gistry; APACHE-HF, **A**cute **P**hysiology **a**nd **C**hronic **H**ealth **E**valuation–**H**eart **F**ailure; EFFECT, **E**nhanced **F**eedback **f**or **E**ffective **C**ardiac **T**reatment; EHMRG, **E**mergency **H**eart Failure **M**ortality **R**isk **G**rade; ELAN-HF, **E**uropean Co**l**laboration on **A**cute Decompe**n**sated **H**eart **F**ailure, EPICTER, registry/study name: **E**studio **p**ara **I**dentificar Pacientes con Insuficiencia **C**ardiaca **Ter**minal (translates roughly as “Study to Identify Patients with Terminal Heart Failure”); ESCAPE, **E**valuation **S**tudy of **C**ongestive He**a**rt Failure and **P**ulmonary Artery Catheterization **E**ffectiveness; GWTG-HF, **G**et **w**ith **t**he **G**uidelines—**H**eart **F**ailure; IPOS-ESC, **I**ntegrated **P**alliative Care **O**utcome **S**cale—**E**uropean **S**ociety of **C**ardiology, MEESSI, **M**ultiple **E**stimation of Risk Based on the Spanish Emergency Department Score in Patients with Acute Heart Failure; MPI, **M**ultidimensional **P**rognostic **I**ndex; OPTIME-CHF, **O**utcomes of a **P**rospective **T**rial of **I**ntravenous **M**ilrinone for **E**xacerbations of **C**hronic **H**eart **F**ailure; PALIAR, (from the Spanish) palliative care program/registry name (not an acronym); PNI-ESC, **P**rognostic **N**utritional **I**ndex – **E**mergency **S**ervices/**C**are; PPI, **P**alliative **P**rognostic **I**ndex; SOB-ASAP, (Components-based acronym): **s**odium, **o**ld age, **b**lood urea nitrogen (B), **a**lbumin, **s**ystolic blood pressure, **a**cute heart failure **p**eptide (BNP/NT-proBNP).

**Table 3:** Variables across the prognostic models

Clinical care area and score name		Variables												
		Age	Functional/mobility status	Vital signs	Cardiac	Renal	Resp	Neuro	Heme	Hepatic	Comorbidities	Symptoms	Treatment/ Medications	Prognostic Awareness
Emergency Department	EHMRG	+	+	+	+	+	+	–	–	–	+		+	–
	SOB-ASAP	+	–	+	+	+	–	–	–	+	–	–	–	–
	MEESSI	+	+	+	+	+	+	–	–	–	–	–	–	–
In-Hospital	GWTG-HF	+	–	+	+	–	+	–	–	–	+	–	–	–
	OPTIME - CHF	+	+	+	+	+	–	–	–	–	–	–	–	–
	ADHERE	-	–	+	+	+	+	–	–	–	–	–	–	–
	EFFECT	+	–	–	+	+	+	+	+	+	+			–
	ESCAPE	+	+	+	+	+	–	–		–	–	–	+	
	ELAN-HF	+	+	+	+	+	–	–	–	–	–	+	–	–
	EPICTER	+	+	–	–	+	+	+	–	–	+		–	+
	In-Hospital Mortality Risk Score	+	+	–	+	+	–	–	+	+	–	–	+	–
	MPI	+	–	+	P	+		–	–	–	–	–	–	–
	PNI-ESC	+	+	–	+	–	–	–	+	–	–	–	–	–
	PALIAR	+	+	–	–	–	–	–	–	+	–	–	–	–
	PPI, Sumi et al.^32^ (2019)	-	–	–	+	–	–	+	–	–	–	–	–	
	Snow et al.^35^ (2016)	+	+	–	+	+	+	+	+	+	+	–	–	–
	IPOS-ESC	-	+	–	–	–	–	–	–	–	–	+	–	+
Critical Care	APACHE-HF	+	–	+	+	+	–	+	+	–	–	–	–	–
	Impact of delirium	+	–	–	+	+	–	+	–	P	P	–	–	–
Multiple Locations	PPI, Martin et al.^34^ (2016)	-	+	–	+	–	–	+	–	–	–	–	–	–

Cardiology.

Palliative Care.

Geriatric Medicine.

+ = Present.

P = Partial.

- = Absent.

Resp = Respiratory Indicators.

Neuro= Neurological Indicators.

Heme= Hematological Indicators.

ADHERE, **A**cute **D**ecompensated **He**art Failure National **Re**gistry; EFFECT, **E**nhanced **F**eedback **f**or **E**ffective **C**ardiac **T**reatment; EHMRG, **E**mergency **H**eart Failure **M**ortality **R**isk **G**rade; APACHE-HF, **A**cute **P**hysiology **a**nd **C**hronic **H**ealth **E**valuation–**H**eart **F**ailure; ELAN-HF, **E**uropean Co**l**laboration on **A**cute Decompe**n**sated **H**eart **F**ailure; EPICTER, registry/study name: **E**studio **p**ara **I**dentificar Pacientes **c**on Insuficiencia Cardiaca **Ter**minal (roughly: “Study to Identify Patients with Terminal Heart Failure”); ESCAPE, **E**valuation **S**tudy of **C**ongestive Heart Failure **a**nd **P**ulmonary Artery Catheterization **E**ffectiveness; GWTG-HF, **G**et **w**ith **t**he **G**uidelines–**H**eart **F**ailure; IPOS-ESC, **I**ntegrated **P**alliative Care **O**utcome **S**cale–**E**uropean **S**ociety of **C**ardiology; MEESSI, **M**ultiple Estimation of Risk Based on the Spanish Emergency Department Score in Patients with Acute Heart Failure; MPI, **M**ultidimensional **P**rognostic **I**ndex; OPTIME-CHF, **O**utcomes of a **P**rospective **T**rial of **I**ntravenous **M**ilrinone for **E**xacerbations of **C**hronic **H**eart **F**ailure; PALIAR, from the Spanish palliative care program/registry name (not an acronym); PNI-ESC, **P**rognostic **N**utritional **I**ndex – **E**mergency **S**ervices/**C**are PPI, **P**alliative **P**rognostic **I**ndex; SOB-ASAP, (Components-based acronym): **s**odium, **o**ld age, **b**lood urea nitrogen (B), **a**lbumin, **s**ystolic blood pressure, **a**cute heart failure **p**eptide (BNP/NT-proBNP).

### Collating, summarizing, and reporting results

Both quantitative and thematic analyses were conducted. Quantitative analyses focused on model performance characteristics, whereas thematic analysis explored patterns in prognostic variables, disciplinary perspectives, and evolution in the models over time. The research team met regularly to develop, define, and refine the prognostic themes within the papers, discussing the utility and conceptualization of prognostication across different disciplines. Informal expert consultation was integrated throughout the review, with cardiology, palliative care, general, and geriatric medicine consultants included on the research team. External consultation from additional cardiology, palliative care, and geriatric medicine clinicians was sought near the end of the process to verify findings and help refine the themes.

## Results

### Prognostic models identified and study characteristics

The review included a heterogeneous body of literature. Smaller and less statistically robust papers were reviewed for context, but after full review, 20 relevant studies were included in the final analysis. These 20 studies were published between 2003 and 2022, and they included 11 studies from the cardiology literature, 5 from the palliative care literature, and 4 from the literature on geriatric medicine.

Study settings included emergency departments, hospital wards, critical care and coronary care units, and outpatient clinics. Most models were derived from hospitalized populations with acute decompensated HF, and a smaller number focused on advanced disease or older adults. HF diagnosis was confirmed clinically,^26^, ^27^, ^28^, ^29^, ^30^, ^31^, ^32^, ^33^, ^34^, ^35^, ^36^, ^37^, ^38^, ^39^ or by Framingham criteria,^40^, ^41^, ^42^, ^43^, ^44^, ^45^ and all studies included adults aged ≥ 18 years. Two papers (SOB- ASAP [**s**odium, **o**ld age, **b**lood urea nitrogen (B), **a**lbumin, **s**ystolic blood pressure, **a**cute heart failure **p**eptide (BNP/N-terminal pro-brain natriuretic peptide {NT-proBNP}], and Multidimensional Prognostic Index [MPI])^37^^,^^45^ were substudies of larger trials. Follow-up timeframes ranged from 1 week^41^ to up to 30 days,^26^^,^^28^^,^^32^^,^^39^^,^^40^^,^^42^^,^^44^ and up to 6 months.^29^, ^30^, ^31^, ^32^, ^33^, ^34^, ^35^, ^36^, ^37^, ^38^^,^^43^ Only 6 studies were explicitly designed to inform palliative care referral or hospice enrollment.^31^, ^32^, ^33^, ^34^, ^35^^,^^37^^,^^44^

#### Descriptive analysis of the cardiology papers

Laboratory values, as prognostic indicators, were almost universal in the papers from the cardiology literature.^26^, ^27^, ^28^^,^^30^^,^^40^, ^41^, ^42^, ^43^^,^^45^ Renal markers (creatinine, blood urea nitrogen, sodium, glomerular filtration rate) appeared in all papers.^26^, ^27^, ^28^^,^^30^^,^^40^, ^41^, ^42^, ^43^^,^^45^ Cardiovascular function (systolic blood pressure, heart rate, troponin) was included in 10 studies,^26^, ^27^, ^28^^,^^30^^,^^40^, ^41^, ^42^, ^43^^,^^45^ and medication use was included in 2 studies.^30^^,^^41^ Other comorbidities and laboratory indicators of multiorgan involvement were common.^28^, ^29^, ^30^, ^31^^,^^40^^,^^41^^,^^43^

Five papers excluded patients with acute coronary syndrome–related HF, palliative approaches, do-not-resuscitate orders, or dialysis dependence. to focus on acute prognostication.^27^^,^^39^, ^40^, ^41^, ^42^ In contrast, the (EPICTER) model (registry/study Name: Estudio para Identificar Pacientes con Insuficiencia Cardiaca Terminal (roughly: “Study to Identify Patients with Terminal Heart Failure”) emphasized functional status, clinician estimated survival of less than 6 months, and acceptance of palliative care.^31^ Although functional and mobility status was not commonly included in the early cardiology prognostication models, it became more prominent beginning in the early 2010s (Table 4). Population sizes of the studies ranged from 423 to 65,275 participants.^26^^,^^29^

**Table 4:** Map of articles grouped by discipline, year (decade), and prognostic variable themes

Years	Cardiology studies, n = 3	Geriatric studies, n = 1	Palliative studies, n = 0
**2000–2009**			
Age	2	1	—
Comorbidities/organ failure	3	1	—
Functional/ mobility status	1	0	—
Prognostic awareness	0	0	—

Years	Cardiology studies, n = 6	Geriatric studies, n = 2	Palliative studies, n = 4
**2010–2019**			
Age	6	2	2
Comorbidities/organ failure	6	2	2
Functional/ mobility status	4	1	4
Prognostic awareness	0	0	0

Years	Cardiology studies, n = 2	Geriatric studies, n = 1	Palliative studies, n = 1
**2020–2025**			
Age	2	1	0
Comorbidities/organ failure	2	1	0
Functional/ mobility status	1	1	1
Prognostic awareness	1	0	1

Values indicate number of papers with key prognostic indicators.

Mortality ranged from 2%-26% at 6 months.^26^, ^27^, ^28^^,^^30^^,^^40^, ^41^, ^42^, ^43^^,^^45^ All papers showed increasing mortality with higher risk strata, with the highest-risk groups reporting 6-month mortality rates ranging from 8.2%-100%.^26^, ^27^, ^28^^,^^30^^,^^40^, ^41^, ^42^, ^43^^,^^45^ Higher reported mortality rates (> 20%) were observed in studies focusing on in-hospital or 30-day mortality and those focusing on advanced or acute decompensated HF^26^^,^^27^^,^^29^^,^^40^^,^^42^^,^^43^ (Table 2).

#### Descriptive analysis of the palliative care papers

Functional status was the central prognostic factor in the palliative care papers, with metrics including independence, nutritional intake, self-care, and mental well-being.^32^, ^33^, ^34^, ^35^^,^^44^ Although organ involvement and the presence of comorbidities were also important prognostic indicators in the palliative care studies, only 2 papers incorporated laboratory values.^32^^,^^35^ Instead, comorbidities and organ involvement were more often indicated in the model by clinical signs and symptoms, such as edema, delirium, and dyspnea.^32^^,^^33^^,^^44^

Early palliative care studies included few HF patients. More recently, palliative care studies started including an increased number of HF patients, reflecting the clinical trend of increasing HF referral rates to palliative and hospice care.^32^^,^^33^ Two studies validated existing prognostic scores in HF populations,^32^^,^^44^ and one developed a new HF-specific tool.^35^ Population sizes of the studies ranged from 294 to 7508 participants.^35^^,^^44^

All palliative models reported 6-month mortality, with overall mortality in the studies in the range of 24%-38%.^32^, ^33^, ^34^, ^35^^,^^44^ Higher risk strata consistently predicted higher mortality^32^, ^33^, ^34^, ^35^^,^^44^ (Table 2).

#### Descriptive analysis of the geriatric medicine papers

Models involving only geriatric patients blended laboratory parameters with clinical signs.^36^, ^37^, ^38^, ^39^ Age was the central theme, with these studies being derived and validated only in older patients.^36^, ^37^, ^38^, ^39^ Renal function markers were common,^36^, ^37^, ^38^, ^39^ and hematologic measures also appeared in some papers.^38^^,^^39^ Functional status was assessed using New York Heart Association (NYHA) class, Katz Activities of Daily Living (ADL) score, or bedridden status.^38^^,^^39^ See the Central Illustration.

**Central Illustration:**
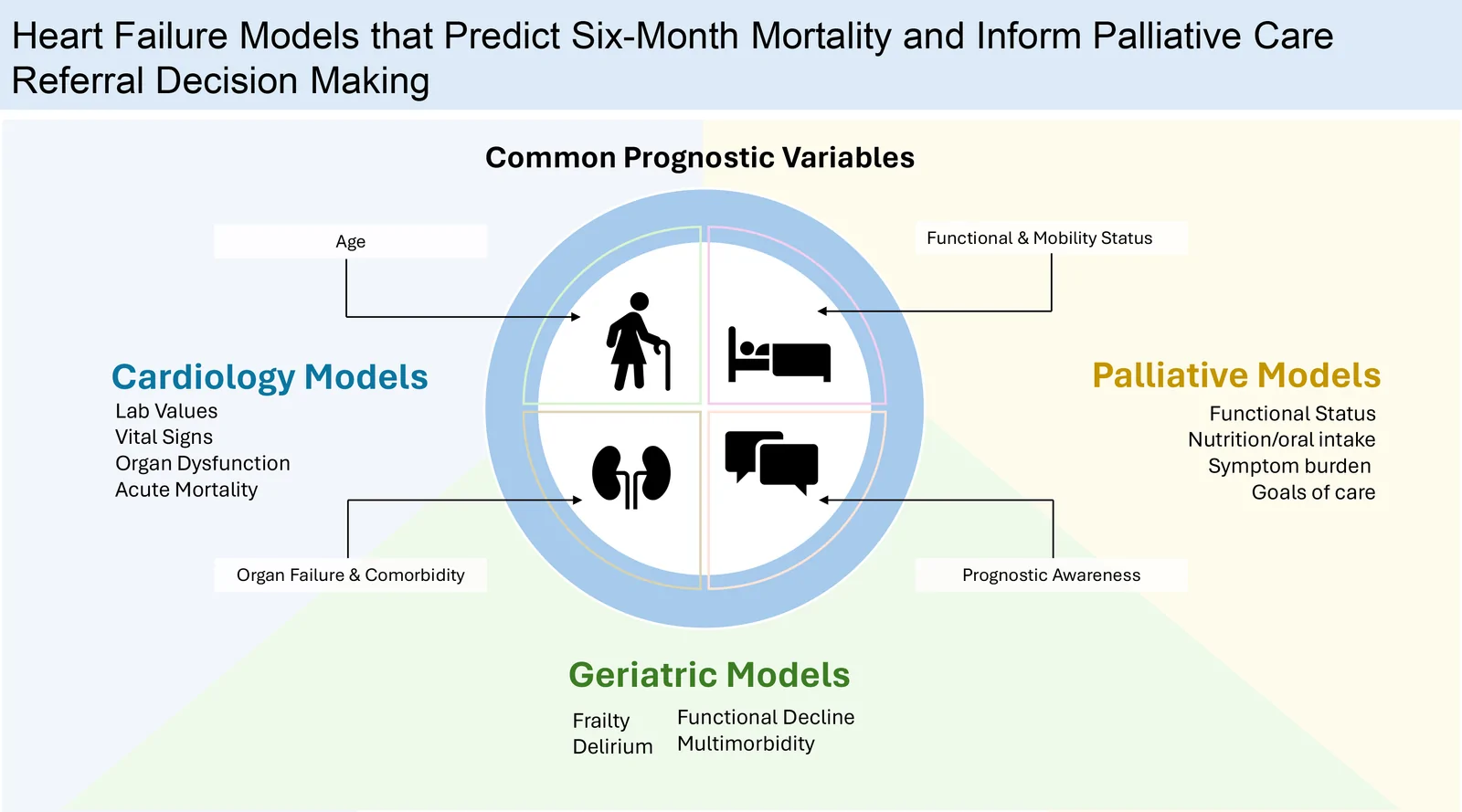
Heart failure models that predict six-month mortality and inform palliative care referral decision-making. This central illustration summarizes the 3 principal disciplines that have developed prognostic models to estimate 6-month mortality in patients with heart failure. The 3 surrounding domains highlight the distinct emphases of cardiology, palliative care, and geriatric models, and the central circle illustrates the convergence of shared prognostic indicators across disciplines. Common variables identified across models include age, functional and mobility status, organ failure and comorbidity burden, and prognostic awareness, reflecting an increasing interdisciplinary alignment in approaches to prognostication and referral planning in advanced heart failure.

Population sizes of the studies ranged from 163 to 629 participants.^36^^,^^39^ Overall 6-month mortality across geriatric medicine papers ranged from 3.9%-41%, with the highest risk–stratified groups having the highest mortality^36^, ^37^, ^38^, ^39^ (see Table 2).

### Overview of reported discrimination across clinical contexts

The performance of the models varied by clinical context and patient population. Overall, cardiology models had larger numbers of participants, and the geriatric models had the smallest numbers. Cardiology-derived tools commonly reported C-statistics in the moderate range with consistent risk stratification across the models’ risk categories.^26^, ^27^, ^28^, ^29^, ^30^, ^31^, ^32^^,^^40^, ^41^, ^42^, ^43^, ^44^, ^45^ Emergency department models were optimized to predict early mortality in patients actively experiencing a crisis, had shorter mortality time frames, and showed higher discrimination (C-statistic ∼0.80-0.83).^41^^,^^42^ These studies may be more applicable to in-hospital palliative care consultation, but less applicable for enrollment in home-based hospice or palliative care programs.

Palliative care models such as the Palliative Prognostic Index (PPI) and the PALIAR score (from the Spanish palliative care program/registry name [not an acronym]) reported modest discrimination (C-statistic ∼0.69-0.72),^33^^,^^34^ but they may be more broadly applicable for a patient population with multiple comorbidities. The MPI, a geriatric model, also reported comparable discrimination (C-statistic ∼0.80) in older, high-comorbidity cohorts, highlighting the prognostic importance of frailty and functional status.^37^

Overall, although cardiology models reported slightly stronger discrimination, especially for predicting very short-term outcomes, palliative and geriatric approaches may provide greater clinical applicability for 6-month prognostication and increasingly multimorbid patient populations, and potentially for triggering timely outpatient palliative care referrals.

### Relevance to palliative referral

When models were evaluated through the lens of palliative care decision-making, substantial variation in clinical applicability emerged. Several HF risk scores demonstrated good discrimination for short-term outcomes but were only indirectly relevant, as they were not designed to identify patients who might benefit from palliative care consultation or hospice referral. In contrast, models such as EPICTER, the PPI, PALIAR, and the MPI were developed or adapted to identify patients with prognoses of short length of life remaining and to help inform palliative care planning.^30^^,^^32^^,^^33^^,^^36^

### Comparative analysis of prognostic indicators

Predictive variables from all the prognostic models could be grouped into the following 4 main categories:

(i) age—defined as an exact number, or an age over a categorical cutoff;
(ii) functional and mobility status—defined by New York Heart Association (NYHA) classification, 6-minute walk test (6MWT) result, bedbound status, Palliative Performance Scale (PPS) score, PPI, Katz ADL score, and arrival by ambulance;
(iii) evidence of comorbidities or organ involvement—defined by vital sign derangement, laboratory abnormalities, clinical diagnoses of other illnesses, high dosage or numbers of medication, and physical signs and symptoms of disease; and
(iv) prognostic awareness—defined by goals of care communication, clinical prediction of survival, and acceptance of palliative care. See the Central Illustration.

#### Age

Age was included in almost all the cardiology models,^27^, ^28^, ^29^^,^^40^, ^41^, ^42^, ^43^^,^^45^ and it was a defining factor in the geriatric medicine models.^36^, ^37^, ^38^, ^39^ However, age was present in only 2 of 5 of the palliative models.^33^^,^^35^ Some models used age as a continuous variable, whereas others used categorical cutoffs (eg, age ≥ 80 or 85 years), reflecting a higher mortality in older age groups.^31^^,^^33^^,^^39^^,^^43^^,^^45^ Although older age is associated with higher mortality, it is not the sole determinant of mortality risk. In geriatric medicine, frailty may more accurately reflect biological vulnerability in older adults, thereby diminishing the independent predictive value of chronological age.^45^, ^46^, ^47^ In palliative care, indicators of advanced disease present as declining functional status, characterized by limited mobility, decreased oral intake, and decreased wakefulness. In very advanced illness, low functional status likely overshadows age as a predictive factor, making age less integral and clinically relevant to the palliative care models.

#### Functional and mobility status

Functional status or mobility metrics appeared in half of the cardiology and geriatric models.^27^^,^^29^^,^^31^^,^^38^^,^^39^^,^^41^^,^^42^ The cardiology and geriatric models most often described functional status according to mobility, with standardized assessment tools, such as the NYHA classification and the 6MWT result.^27^^,^^29^^,^^31^^,^^38^^,^^39^^,^^42^ Two additional papers assessed mobility by including the need for transportation by emergency medical services or bedridden status.^38^^,^^41^ Geriatric models more often incorporated multidimensional assessments, including ADLs and multiple functional measures.^38^^,^^39^ In palliative care, functional status was universal.^32^, ^33^, ^34^, ^35^^,^^44^ In addition to mobility, all palliative models included nutritional status and oral intake, reflecting the importance of nutrition, metabolism, and wakefulness on functional status in advanced disease.^32^^,^^33^^,^^44^

#### Comorbidities/secondary organ involvement

The presence of comorbidities and/or secondary organ involvement was a prognostic indicator common to all models and across all disciplines. These prognostic indicators are presented individually in Table 3. These indicators can also be thought of together, though this makes a very heterogeneous group, as it is often not possible to determine whether specific signs or symptoms are due to advanced HF or to another comorbidity. This group of heterogenous indicators reflects the reality of current HF populations, in which multimorbidity is increasingly common and must be considered when assessing prognosis and potential palliative care needs. Conceptually, combining these indicators is relevant for end-of-life prognostication, where the labelling of what illness, or laboratory abnormality, is causing a decline is often less clinically relevant for the patient than the impact of the decline itself.

Cardiology papers defined comorbidities and secondary organ involvement with specific laboratory value cutoffs, associated with an increased risk of mortality.^26^^,^^27^^,^^40^, ^41^, ^42^, ^43^, ^44^, ^45^ End-stage organ involvement and comorbidities were reflected in the palliative scores, but instead were based on signs and symptoms, such as level of consciousness, delirium, edema, and dyspnea.^32^, ^33^, ^34^, ^35^^,^^44^ The geriatric medicine papers used cardiovascular and renal function markers as predictors, but also had a greater focus on anemia, hypoalbuminemia, dementia, and delirium.^36^, ^37^, ^38^, ^39^ Medications were listed as a prognostic indicator in 3 papers analyzed.^29^^,^^39^^,^^41^ The medications were often related to the underlying HF, but theycan also serve as surrogate markers for comorbid conditions or secondary organ involvement.

A continuum in how these indicators were handled in the 3 disciplines was identified. The cardiology models focused on organ-specific dysfunction that was often indicated and/or diagnosed by laboratory values. However, as patients progress in age (geriatric populations) or toward the end of life (palliative populations), the emphasis becomes more on the number and severity of the illnesses, and finally on the impact of the combined illnesses on the individual patient. Comorbidities and organ involvement identified in all the disciplines impacted survival, but when the diagnoses are made with laboratory values, they are identified earlier than when they are diagnosed clinically. This difference is reflected in the increasing overall mortality rates in cardiology models compared to geriatric medicine models and again compared with the palliative models (Table 1).

#### Prognostic awareness

Holistic domains, such as symptom burden, communication, and goals of care are infrequently included, and except for in EPICTER,^31^ these domains are found only in geriatric and palliative frameworks. Unique to EPICTER, clinical prediction of a survival rate of < 6 months, estimated by the treating physician, and acceptance of palliative care, was found to be useful in the estimation of 6-month mortality.^31^ **I**ntegrated **P**alliative Care **O**utcome **S**cale European Society of Cardiology (IPOS-ESC) emphasized holistic domains such as communication, emotional support, and practical care needs.^44^ Although clinical predictions of survival and well-being/acceptance are predictive, they may be challenging for some care providers to assess and quantify. This difficulty may result in limited provider buy-in and ongoing provider preferences to use other prognostic models that they may regard as more familiar or more objective (see Table 3 and Central Illustration).

### Convergent evolution in prognostic models over time

A comparison of the articles from all the disciplines over time, reveals a gradual evolution in the prognostic models. After the 2000s, the cardiology papers begin to include more aspects of functional and mobility assessment, and the palliative care studies start to validate and create their scores in HF-specific populations (Table 4). A gradual shift seems to be occurring within the specialty-specific models as they incorporate additional prognostic factors from the other disciplines to better assess the unique, and increasingly complex, needs of end-of-life HF patients. This convergence in evolution is resulting in more clinically balanced approaches to prognostication, responding to the increasing complex needs of an aging and more comorbid advanced HF population over time. (Central Illustration).

## Discussion

In this scoping review, we identified 20 studies across various settings that could be used to help inform clinicians about the potential timing for palliative care referrals in patients with HF. This review showed that no one predictive model had obvious superiority, leaving the choice of which to use up to the individual care provider, with potential influence from the care setting.

Across disciplines, prognostic indicators existed along a continuum. Cardiology relied more heavily on laboratory markers, whereas geriatrics and palliative care emphasized multimorbidity and functional decline. Over time, models increasingly converged around age, comorbidity and/or organ dysfunction, functional status, and holistic domains.

Cardiology tools that rely heavily on laboratory data may exclude patients who decline further investigations in favour of a palliative approach, and geriatric medicine models do not address younger patients who might be eligible for advanced therapies, such as ventricular assist devices or transplantation. In contrast, palliative care models rely heavily on functional assessments that may be less familiar, less explicitly incorporated, or less heavily weighted, in prognostication by clinicians outside the discipline. In addition, use of palliative care models that use functional assessments without accompanying laboratory measures may be less comfortable for some clinicians who prefer to have laboratory and imaging findings to support their clinical predictions of survival.

Identifying patients at risk of death within 6 months remains challenging given prognostic uncertainty, care fragmentation, fluctuating patient preferences, and changing functional status, all of which may delay goals-of-care discussions and palliative care referrals.

Ideally, discussions about goals of care should be held early, should be ongoing, and should be nonthreatening. These factors help ensure that patient-centred goals continue to be met throughout the illness trajectory. However, responsibility for these discussions is unclear. Among cardiologists, 66% believe these conversations should be done by primary care providers, whereas 57% of primary care providers believe they should be done by cardiology providers.^7^^,^^10^ This situation leaves many patients without timely goals-of-care discussions or access to additional support. Although prognostic uncertainty is only one barrier, improved understanding of end-of-life prognostication may facilitate these conversations.

A key finding was the relative immaturity of the evidence base supporting prognostic models for palliative care referral in HF. Despite more than 2 decades of research, studies remain highly heterogeneous with respect to patient populations, prognostic horizons, clinical settings, and model construction. This fragmentation has resulted in numerous modestly predictive tools but no widely accepted approach to identifying patients who may benefit from palliative care or hospice referral.

This finding highlights an opportunity to explore whether a unified prognostic approach that integrates the most predictive variables across disciplines could improve clinician confidence in initiating end-of-life discussions and support timely, appropriate palliative care consultation and hospice referrals.

### Limitations

This study has several limitations. As is common in scoping reviews, one possibility is that not all relevant articles have been included. Although a comprehensive search was performed across multiple databases, the search strategy was developed to identify studies relevant to 6-month mortality and palliative care referral decision-making, rather than to identify every published HF prognostic model. Consequently, some historically important prognostic models may not have been captured. In addition, the iterative nature of the search strategy development may limit reproducibility, as search teams and eligibility criteria were refined during the review process. Some eligibility decisions required reviewer judgement, particularly when mortality was a secondary outcome or multiple prognostic endpoints were reported. Although criteria were applied consistently by 2 reviewers, some subjectivity was unavoidable. This limitation is inherent to the scope and objectives of the review.

Multiple other well known and well regarded cardiology prognostic scoring systems were not included because they had prognostic end points of 1 year or more. Similarly, some palliative care papers were excluded due to classification of patients as having “heart disease,” “cardiac disease,” or “non-cancerous conditions,” and one palliative care paper that specifically included patients with HF was omitted, because the population included only patients who had already been admitted to an outpatient hospice program.^48^ These omitted scoring systems likely also included patients with HF who died within 6 months, and these papers might have contributed to the knowledge on the subject. We also note that over a year has elapsed since the literature search was completed, which may mean that very novel prognostication models were missed.

Another major limitation in our scoping review was the significant heterogeneity among included articles, which made direct comparisons challenging, and thereby possibly had impact on the reliability of our conclusions. For example, the scoring systems reviewed predicted different timelines for mortality, with some studies predicting death within days^26^^,^^28^^,^^32^^,^^39^, ^40^, ^41^, ^42^^,^^45^ and others predicting death within 6 months.^29^, ^30^, ^31^, ^32^, ^33^, ^34^, ^35^, ^36^, ^37^, ^38^^,^^43^^,^^44^ This difference is particularly evident in the cardiology papers, which have a wide range of overall mortalities due to diversity in study design and setting. Considerable variation was also present in participant characteristics, study design, and setting that is likely to have affected prognosis. In addition, model performance was limited by inconsistent reporting of calibration measures across studies, which restricted assessment of overall predictive accuracy. This heterogeneity limited direct comparison of model performance and reinforced the need for a thematic synthesis rather than a quantitative comparison across studies.

## Conclusion

Current prognostic scoring systems offer some utility in estimating prognosis in advanced HF, but they remain suboptimal for identifying patients appropriate for hospice and palliative care referral. This review shows that a wide range of very ill HF populations have been studied using variable approaches, resulting in multiple, modestly predictive models for predicting mortality up to 6 months. We concluded that although each study had unique strengths and limitations, the recommendation of which model(s) to use is both clinician- and context-dependent.

Although cardiology, palliative care, and geriatric medicine have each developed valuable models, and they predict mortality in HF populations, the models are quite different, with different locations and contexts of care. The different contexts and variables reflect how prognostication is viewed differently across the disciplines, reflecting different priorities on how prognostication affects clinical care. This review highlights both the overlap and differences in prognostic variables across these models and provides clinicians with a range of tools to consider in practice. The review also demonstrates an increasing convergence of key prognostic factors across disciplines over time.

These findings suggest an important opportunity, and need, for further study in large, inclusive inpatient populations, to evaluate whether combining variables across existing models can improve prediction of 6-month mortality. Future prognostic tools should integrate the most relevant predictors from across the disciplines, explore new variables, and ensure that model indicators remain feasible to collect and are minimally burdensome for patients and care providers.

## References

[1] Heart and Stroke Foundation of Canada Falling short: how Canada is failing people with heart failure—and how we can change that. 2022 Spotlight on Heart Failure. https://www.heartandstroke.ca/-/media/pdf-files/canada/2022-heart-month/hs-heart-failure-report-2022-final.pdf.

[2] (2018). Global Burden of Disease 2017 disease and injury incidence and prevalence collaborators. Global, regional, and national incidence, prevalence, and years lived with disability for 354 diseases and injuries for 195 countries and territories, 1990-2017: a systematic analysis for the Global Burden of Disease Study 2017. Lancet.

[3] Steinberg L., White M., Arvanitis J., Husain A., Mak S. (2017). Approach to advanced heart failure at the end of life. Can Fam Phys.

[4] Sobanski P.Z., Alt-Epping B., Currow D.C. (2020). Palliative care for people living with heart failure: European Association for Palliative Care Task Force expert position statement. Cardiovasc Res.

[5] Poon S., Leis B., Lambert L. (2022). The state of heart failure care in Canada: minimal improvement in readmissions over time despite an increased number of evidence-based therapies. CJC Open.

[6] Park J.S., Seo K.W., Lee J.E., Kim K.H., Ahn J.A. (2025). Communication needs regarding heart failure trajectory and palliative care between patients and healthcare providers: a cross-sectional study. PLoS One.

[7] Goodlin S., Quill T., Arnold R. (2008). Communication and decision-making about prognosis in heart failure care. J Card Fail.

[8] Lunney J.R., Lynn J., Foley D.J., Lipson S., Guralnik J.M. (2003). Patterns of functional decline at the end of life. JAMA.

[9] Gelfman L.P., Sudore R.L., Mather H. (2020). Prognostic awareness and goals of care discussions among patients with advanced heart failure. Circ Heart Fail.

[10] Quinn K.L., Hsu A.T., Smith G. (2002). Association between palliative care and death at home in adults with heart failure. J Am Heart Assoc.

[11] Levy W.C., Mozaffarian D., Linker D.T. (2006). The Seattle Heart Failure Model: prediction of survival in heart failure. Circulation.

[12] Pocock S.J., Ariti C.A., McMurray J.J.V. (2013). Predicting survival in heart failure: a risk score based on 39 372 patients from 30 studies. Eur Heart J.

[13] Wiskar K., Toma M., Rush B. (2018). Palliative care in heart failure. Trends Cardiovasc Med.

[14] Liu A.Y., O'Riordan D.L., Marks A.K., Bischoff K.E., Pantilat S.Z. (2020). A comparison of hospitalized patients with heart failure and cancer referred to palliative care. JAMA Netw Open.

[15] Heidenreich P.A., Bozkurt B., Aguilar D. (2022). 2022 AHA/ACC/HFSA guideline for the management of heart failure: a report of the American College of Cardiology/American Heart Association Joint Committee on Clinical Practice Guidelines. Circulation.

[16] Bhattacharya A., Chakrabarty S., Cabrales J. (2023). Implementation of a palliative care consultation trigger tool for hospitalized patients with acute decompensated heart failure. BMJ Open Qual.

[17] (2025). Canadian Virtual Hospice, How is eligibility for palliative care decided? What procedure is followed?. https://www.virtualhospice.ca/en_US/Main+Site+Navigation/Home/Support/Support/Asked+and+Answered/Palliative+Care/How+is+eligibility+for+palliative+care+decided_+What+procedure+is+followed_.aspx.

[18] Wagner V., Marks A. (2024). What is hospice?. JAMA.

[19] Tricco A.C., Lillie E., Zarin W. (2018). PRISMA extension for scoping reviews (PRISMA-ScR): checklist and explanation. Ann Intern Med.

[20] Arksey H., O’Malley L. (2005). Scoping studies: towards a methodological framework. Int J Soc Res Methodol.

[21] Levac D., Colquhoun H., O’Brien K.K. (2010). Scoping studies: advancing the methodology. Implement Sci.

[22] Munn Z., Peters M.D.J., Stern C. (2018). Systematic review or scoping review? Guidance for authors when choosing between a systematic or scoping review approach. BMC Med Res Methodol.

[23] Maestre A., Gil V., Gallego J. (2009). Diagnostic accuracy of clinical criteria for identifying systolic and diastolic heart failure: cross-sectional study. J Eval Clin Pract.

[24] Moons K.G.M., Wolff R.F., Riley R.D. (2019). PROBAST: a tool to assess risk of bias and applicability of prediction model studies: explanation and elaboration. Ann Intern Med.

[25] Moons K.G., Altman D.G., Reitsma J.B. (2015). Transparent reporting of a multivariable prediction model for individual prognosis or diagnosis (TRIPOD): explanation and elaboration. Ann Intern Med.

[26] Fonarow G.C., Adams K.F., Abraham W.T., Yancy C.W., Boscardin J.W. (2005). Risk stratification for in-hospital mortality in acutely decompensated heart failure. JAMA.

[27] Felker G.M., Leimberger J.D., Califf R.M. (2004). Risk stratification after hospitalization for decompensated heart failure. J Card Fail.

[28] Peterson P.N., Rumsefeld J.S., Liang L. (2010). A validated risk score for in-hospital mortality in patients with heart failure from the American Heart Association Get with the Guidelines Program. Circ Cardiovasc Qual Outcomes.

[29] Salah K., Stienen S., Moons A.H.M. (2019). External validation of the ELAN-HF score: predicting 6-month all-cause mortality in patients hospitalized for acute decompensated heart failure. J Am Heart Assoc.

[30] O’Connor C.M., Hasselblad V., Mehta H. (2010). Triage after hospitalization with advanced heart failure: the ESCAPE (Evaluation Study of Congestive Heart Failure and Pulmonary Artery Catheterization Effectiveness) risk model and discharge score. JACC.

[31] Romero-Correa M., Alamanca-Bautista P., Bilbao-Gonzalez A. (2022). The EPICTER score: a bedside and easy tool to predict mortality at 6 months in acute heart failure. Eur Soc Card Heart Fail J.

[32] Sumi T, Umemoto N, Kajiura H (2019). P4551. Prognostic utility of Palliative Prognostic Index for prediction of 30-day and 1-year outcome in patients with acute decompensated heart failure. Eur Heart J.

[33] Bernabeu-Wittel M., Murcia-Zaragoza J., Hernandez-Quiles C. (2014). Development of a six-month prognostic index in patients with advanced chronic medical conditions: the PALIAR score. J Pain Symptom Manage.

[34] Martin N., Bernabeu-Wittel M., de la Higuera V.L. (2013). Adaptation of the palliative prognostic index in patients with advanced medical conditions. Rev Clin Esp (Barc).

[35] Snow R., Vogel K., Vanderhoff B., Kelch B.P., Ferris F.D. (2016). A prognostic indicator for patients hospitalized with heart failure. J Pall Med.

[36] Sato K., Kubota K., Oda H., Taniguchi T. (2017). The impact of delirium on outcomes in acute, non-intubated cardiac patients. Eur Heart J Acute Cardiovasc Care.

[37] Huynh B.C., Rovner A., Rich M.W. (2008). Identification of older patients with heart failure who may be candidates for hospice care: development of a simple four-item risk score. J Am Geriatr Soc.

[38] Candeloro M., Di Nsio M., Balducci M. (2020). Prognostic nutritional index in elderly patients hospitalized for acute heart failure. Eur Soc Card Heart Fail.

[39] Abarca J.M., Arana L.F., Martin-Sanchez F.J. (2018). In hospital mortality risk score for very old patients hospitalized with decompensated chronic heart failure. Eur Geriatr Med.

[40] Lee D.S., Austin P.C., Rouleau J.L. (2003). Predicting mortality among patients hospitalized for heart failure: derivation and validation of a clinical model. JAMA.

[41] Lee D.S., Stitt A., Austin P.C. (2012). Prediction of heart failure mortality in emergent care. Ann Intern Med.

[42] Wussler D., Kozhuharov N., Sabti Z. (2019). External validation of MEESSI acute heart failure risk score: a cohort study. Ann Intern Med.

[43] Okazaki H., Shirakabe A., Hata N. (2014). New scoring system (APACHE-HF) for predicting adverse outcomes in patients with acute heart failure: evaluation of the APACHE II and modified APACHE II scoring systems. J Cardiol.

[44] Hamatani Y., Iguchi M., Ikeyama Y. (2022). Comprehensive symptom assessment using integrated palliative care outcome scale in hospitalized heart failure patients. Eur Soc Cardio Heart Fail J.

[45] Shiraishi Y., Kohaska S., Abe T. (2020). Derivation and validation of clinical prediction models for rapid risk stratification for time-sensitive management for acute heart failure. J Clin Med.

[46] Rockwood K., Song X., MacKnight C. (2005). A global clinical measure of fitness and frailty in elderly people. CMAJ.

[47] Fried L.P., Tangen C.M., Walston J. (2001). Frailty in older adults: evidence for a phenotype. J Gerontol A Biol Sci Med Sci.

[48] Creber R.M., Russell D., Dooley F. (2019). Use of the palliative performance scale to estimate survival among home hospice patients with heart failure. Eur Soc Card Heart Fail.

